# Can party balloons replace autoinflation balloons to treat glue ear? A technical comparison

**DOI:** 10.3399/BJGPO.2020.0178

**Published:** 2021-04-21

**Authors:** Katherine Marshall, Rafael Perera, Paul Glasziou, Susannah Fleming

**Affiliations:** 1 Medical School, University of Oxford, Oxford, UK; 2 Nuffield Department of Primary Care Health Sciences, University of Oxford, Oxford, UK; 3 Faculty of Health Sciences & Medicine, Bond University, Robina, QLD, Australia

**Keywords:** otitis media with effusion, Valsalva maneuver, pediatrics, primary health care, general practice

## Abstract

**Background:**

Autoinflation balloons are used to treat patients with otitis media with effusion (OME) to help avoid surgery.

**Aim:**

To compare the ability of party balloons with Otovent balloons to produce sufficient pressure for a Valsalva manoeuvre.

**Design & setting:**

Pressure testing was used to determine the number of times each balloon could produce pressures sufficient for a Valsalva manoeuvre. Subsequently, Otovent balloons were compared with spherical party balloons in a pilot clinical trial of 12 healthy adults.

**Method:**

Each balloon was inflated 20 times and the maximum pressure was recorded. Three balloons of each type were tested to 50 inflations to assess pressures over persistent use.

**Results:**

Otovent balloons’ mean inflation pressure was 93 mmHg (95% confidence interval [CI] = 89 to 97 mmHg) on first inflation, dropping to 83 mmHg (95% CI = 80 to 86 mmHg) after 20 inflations. Two types of spherical party balloon required mean inflation pressures of 84 mmHg (95% CI = 77 to 90 mmHg) and 108 mmHg (95% CI = 97 to 119 mmHg) on first inflation, dropping to 74 mmHg (95% CI = 68 to 81 mmHg) and 83 mmHg (95% CI = 77 to 88 mmHg) after 20 inflations. In the pilot trial, there was no difference between the ability of Otovent and spherical balloons (χ^2^ = 0.24, *P* = 0.89) to produce the sensation of a Valsalva manoeuvre.

**Conclusion:**

Otovent balloons can be used more than the 20 times quoted by the manufacturer. The two spherical balloons produced similar pressures to Otovent balloons, indicating potentially the same clinical effect. The pilot study suggests a potential use of spherical party balloons instead of Otovent balloons as a cost-efficient treatment.

## How this fits in

Autoinflation balloons, such as Otovent, can be used as an effective treatment option in OME. This research shows that two types of spherical party balloons are able to produce similar pressures to Otovent balloons. It also shows that both Otovent and spherical party balloons are capable of producing sufficient pressure for a Valsalva manoeuvre for at least 50 inflations. This provides the potential for reduced cost autoinflation treatment for children with OME.

## Introduction

OME, commonly known as glue ear, is a non-inflammatory fluid blockage in the middle ear,^1^ without symptoms of acute infection. Grommet insertion to treat glue ear is the most common reason for a surgical procedure in childhood in the UK, with 25 000 procedures performed each year.^2^ OME has a point prevalence of 20% in 2 year olds,^3^ and by the age of 4 years has a cumulative incidence of 80%.^4^ OME can lead to conductive hearing loss; for patients with bilateral OME for over a month, this can be as great as 20–30dB.^5^ This hearing loss resolves in 95% of cases within a year,^6^ although patients may experience linguistic, developmental, and behavioural consequences.^7^ Current National Institute for Health and Care Excellence (NICE) guidelines suggest a 3-month period of watchful waiting^8^ as OME has been shown to resolve in 50% of cases during this period.^9^ Furthermore, in children aged <3 years, a delay of 6 months in surgical treatment does not appear to significantly affect developmental outcomes.^10^ Medical treatment options include mucolytics, antihistamines, corticosteroids, decongestants,^11^ and antibiotics.^12^ Antibiotic treatments show either short-lived benefit for 1 month^13^ or no benefit at all.^14^ Corticosteroids can be effective in the short term,^15^ although no long-term benefit has been shown.^16^

Autoinflation balloons can be used to treat OME, as shown in a recent systematic review,^17^ with a number needed to treat of 9.^18^ These balloons equalise the pressure between the middle ear and nasal cavity. The intranasal pressure is increased on inflation of the balloon, forcing open the eustachian tube and equalising the negative pressure. This process is a Valsalva manoeuvre,^19^ with 40 mmHg usually quoted for an effective Valsalva manoeuvre (range of 30–50 mmHg).^20–24^ The balloon provides a visual aid to help the patient complete a Valsalva manoeuvre. Otovent balloons are a type of autoinflation balloon sold as a set of five with a nosepiece. The balloon is fitted to the flat end of the nosepiece while the ball-shaped section is placed to form a tight seal against the nostril; the other nostril is compressed.^25^ Manfacturers of Otovent balloons recommend a maximum of 20 inflations per balloon, so Otovent balloons were tested to determine after how many repeated inflations they could produce a pressure of 40 mmHg. Four different types of party balloons were also tested to determine whether they could reach adequate pressures to be used as a potential alternative method for the treatment of OME.

## Method

**Figure 1.**
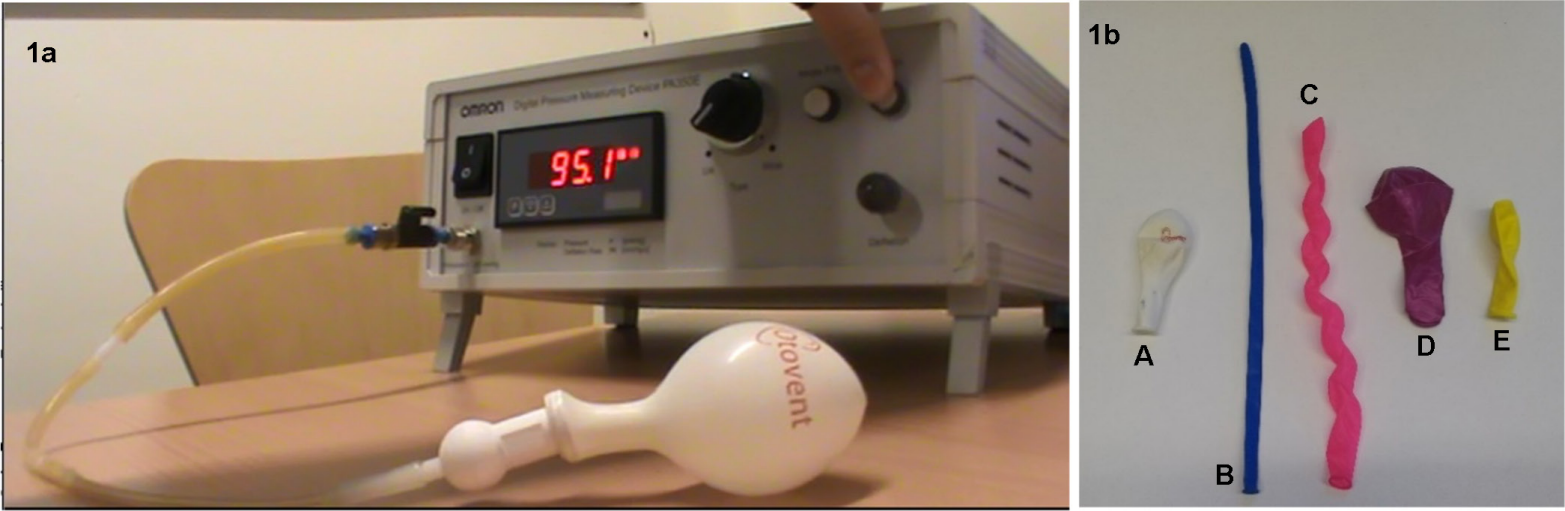
Test equipment used in the study. (Figure 1a) Omron PA350e blood pressure monitor used to test the maximum pressure achieved by each balloon, connecting tubes, nosepiece, and Otovent balloon. (Figure 1b) Balloons tested, left to right: Otovent (A), modelling (B), spiral (C), sphere 1 (D), sphere 2 (E)

A novel protocol was designed using the Omron PA350e blood pressure monitor tester to inflate the balloons and measure the pressure (Figure 1a). The pressure-testing device has previously been used to assess the accuracy of sphygmomanometers used in primary care.^26^ This apparatus consisted of three pieces of rubber tubing linked together by connectors, with one end attached to the nosepiece designed for Otovent balloons.^25^ To inflate the balloon, air was pumped through the plastic tubing into the balloon allowing the pressure within to be measured.

As the balloon inflated, the pressure within rose until a critical point when the volume of air within the balloon continued to increase but the pressure decreased. After the maximum pressure had been reached by each balloon (shown by a decline in pressure despite an increase in volume) the machine was turned off, allowing the balloon to deflate. This process was repeated 20 times unless the balloon burst, as this is the limit quoted by Otovent. Each inflation was video-recorded, enabling the digital display of the machine to be checked to determine the maximum pressure reached by each balloon.

Five different types of balloons were tested (Figure 1b); eight Otovent balloons (A), and 10 party balloons of each of four different types (modelling [B], spiral [C], sphere 1 [D], and sphere 2 [E]). The party balloons selected were easily available and included a range of shapes, sizes, and manufacturers. The modelling balloons were long and thin, while the spiral balloons were long and irregularly shaped. The Otovent (A) and sphere 2 (E) balloons were small and spherical; sphere 1 (D) balloons were larger in size but had the same shape. All available colours of balloons were tested to give an initial indication as to whether colour affected the maximum pressure of the balloon.

The spiral balloons, owing to their irregular shape, required a maximum pressure to be recorded after 5 seconds as well as after 30 seconds. This allowed the pressures within the balloon to be acurrately determined throughout the course of the 20 inflations. Four of the 10 modelling balloons did not inflate despite being pressurised to 300 mmHg (the maximum pressure produced by the testing device) and, therefore, were removed from the analysis.

In addition, three of each of the Otovent (A), sphere 1 (D), and sphere 2 (E) balloons were subjected to an extended test of 50 inflations to determine if they were still able to achieve the pressure of 40 mmHg.

The data for each inflation number for each balloon was summarised by mean and CIs. Linear regression was then used to analyse the data. These equations were used to extrapolate the data until the point at which a Valsalva manoeuvre could no longer occur (maximum pressure of 40 mmHg). Following this, a linear mixed-effects model was used with both varying intercept and varying slope, allowing for the original grouping of the data by individual balloon. This model allowed variability between different balloons of the same type to be determined. Furthermore, this data was used to determine the longevity of the ‘worst predictable’ balloon. This was determined by finding the mean minus 2SD for the intercept and slope. All CIs are 95%, and results were considered statistically significant if the *P* value was <0.05. Analyses were performed in R (version 3.3.1).

### Pilot clinical trial

Following the successful tests of both spherical balloons (D and E), a pilot clinical trial was carried out to determine their effectiveness in ability to perform a Valsalva manoeuvre in comparison with Otovent balloons (A). Participants were healthy, aged 18–65 years and recruited via word of mouth. Exclusion criteria included having history of recent nasal trauma or bruising; severe coronary artery disease; a moderate to severe reduction in blood volume; or having experienced a recent heart attack. Informed consent was achieved by a verbal explanation of the study at the time of consent as well as a participant information sheet; 12 participants gave their written informed consent.

Participants were randomised into three groups and used the balloons in three different orders (ADE, DEA, and EAD, respectively). It was not possible to use a placebo or to double-blind the trial by masking which balloons were being used. Each participant was asked to inflate the three balloons in turn by placing the balloon to one nostril, covering the other, and breathing out into the balloon; it was recorded whether they felt their ear 'pop'. Inflation was carried out without a nosepiece for all balloons. If this occurred, a Valsalva manoeuvre was deemed to have successfully taken place. A successful Valsalva manoeuvre was recorded as either a ‘yes’ or ‘no’ to ear popping. The results were analysed by finding the percentage success rate and followed by a χ^2^ test of independence to compare the groups.

## Results

**Figure 2.**
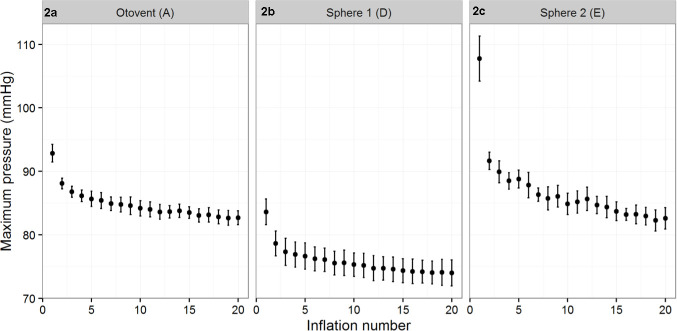
Mean inflation pressures (2–20) and standard deviation of (Figure 2a) Otovent (A), *n* = 8; (Figure 2b) sphere 1 (D), *n* = 10; (Figure 2c) sphere 2 (E), *n* = 10.

Table 1 shows the mean inflation pressures and CIs for all balloons at initial inflation; after 20 inflations (Figure 2, supplementary Figures S2 and S3); and, where relevant, after 50 inflations (Figure S1). The modelling balloons (B) were found to require excessively high inflation pressures. The long spiral balloons (C) had an unpredictable and variable pressure response during inflation, with two balloons bursting, making their results unreliable. The Otovent balloons (A) achieved the required pressure throughout testing, along with both types of spherical party balloon (D and E). All of these balloons showed linear decreases in the pressure required to inflate them from the second inflations.

**Table 1.** Mean and 95% confidence intervals of maximum pressures (mmHg) achieved during the first 30 seconds of inflation for each balloon type

**Balloon type**		**Initial inflation**	**After 20 inflations**	**After 50 inflations**
*n*	Mean (mmHg)	95% confidence interval **(mmHg)	Mean (mmHg)	95% confidence interval **(mmHg)	Mean (mmHg)	95% confidence interval **(mmHg)
Otovent (A)	8	92.9	88.9 to 96.8	82.7	79.6 to 85.7	77.4	74.0 to 80.8
Modelling (B)	10	278.1	221.14 to 335.0	107.5	75.7 to 139.2	n/a	n/a
Spiral – 5 seconds (C)	10	79.77	70.6 to 89.0	58.5	53.6 to 63.4	n/a	n/a
Spiral – 30 seconds (C)	10	74.77	64.6 to 84.8	62.1	57.5 to 66.7	n/a	n/a
Sphere 1 (D)	10	83.6	77.3 to 90.0	74.0	67.5 to 80.5	71.2	66.5 to 75.9
Sphere 2 (E)	10	107.7	96.5 to 118.9	82.6	77.3 to 87.9	77.1	67.8 to 86.4

### Linear regression analysis

Sphere 1 (D) had the shallowest gradient closely followed by Otovent (A) and then sphere 2 (E). Sphere 2 (E) had the highest intercept, before Otovent (A) and then sphere 1 (D) (supplementary Figure S4). The results were extrapolated to predict how many times the balloons could be inflated before they were no longer able to carry out a Valsalva manoeuvre (Table 2).

**Table 2.** Linear regression results (inflation number 2–50) for Otovent (A), sphere 1 (D), and sphere 2 (E)

**Balloon**	***n***	**Equation**	**CI intercept**	**CI gradient**	***P* value of gradient**	**Predicted number of inflations** **when maximum pressure is 40** mmHg
Otovent (A)	8	Y=–0.1775*X+86.63	86.23 to 87.02	–0.191 to –0.1641	<0.0001	262
Sphere 1 (D)	10	Y=–0.1108*X+77.13	76.49 to 77.77	–0.1323 to –0.0893	<0.0001	335
Sphere 2 (E)	10	Y=–0.2355*X+88.70	88.05 to 89.34	–0.2574 to –0.2136	<0.0001	206

### Mixed-effect model

To analyse the variability between different balloons of the same type, a mixed-effect model was used. Sphere 2 (E) had the highest intercept followed by Otovent balloons (A) and sphere 1 (D), as shown in Table 3. Sphere 1 (D) had the shallowest gradient, followed by Otovent (A) and then sphere 2 (E). When comparing the ‘worst predictable balloon’ model, Otovent (A) balloons showed the least variability and had the highest number of inflations before being unable to complete a Valsalva manoeuvre (maximum pressure dropped below 40 mmHg). Sphere 1 (D) balloons lasted for more inflations than sphere 2 (E) balloons, but both had a lower maximum inflation number than the Otovent (A) balloon. A ‘worst predictable balloon’ model was calculated using mean-2SD for both the intercept and slope.

**Table 3.** Mixed-effect model for inflation numbers 2–50: results and inflation number of 'worst predictable' balloon for Otovent (A), sphere 1 (D), and sphere 2 (E).

**Balloons**		**Intercept**	**Slopes**	**‘Worst predictable balloon’** **inflation number when maximum pressure is 40** mmHg
***n***	**Mean**	**SD**	**Mean**	**SD**
Otovent (A)	8	86.75	1.446	–0.204	0.0163	185
Sphere 1 (D)	10	77.50	3.384	–0.18	0.0866	87
Sphere 2 (E)	10	90.20	2.377	–0.402	0.156	63

### Pilot clinical study results

Participants had an equal success rate with Otovent balloons (A) and sphere 2 (E) balloons (Table 4). The χ^2^ test showed no difference between the three balloon groups (χ^2^ = 0.2408, *P* = 0.887).

**Table 4.** Success rates of Otovent (A), sphere 1 (D), and sphere 2 (E) in causing a Valsalva manoeuvre when inflated (*n* = 12)

**Balloons**		**Success rate**
***n***	Mean (%)	95% confidence interval (%)
Otovent (A)	12	66.7	40.0 to 93.3
Sphere 1 (D)	12	58.3	30.4 to 86.2
Sphere 2 (E)	12	66.7	40.0 to 93.3

## Discussion

### Summary

Overall, it has been shown that Otovent balloons can be used more than the 20 times printed on the manufacturer’s guidelines, and will still be able to produce a Valsalva manoeuvre after at least 50 uses. Furthermore, it has been shown that spherical party balloons are able to produce similar pressures for a similar number of inflations. Balloon shape is an important factor in the use of party balloons, as neither the modelling nor spiral balloons were effective owing to the long and either irregular or thin shape. As the current NICE guidelines^8^ suggest a period of watchful waiting for the first 3 months before any surgical treatment, the use of spherical party balloons could provide a cost-efficient treatment option during this time period. Extrapolating the linear regression and mixed-model data suggests that it would take over nine times the manufacturer’s limit of 20 inflations before Otovent balloons were no longer able to reach the necessary pressure. However, party balloons (sphere 1 [D] and sphere 2 [E]) are still able to be inflated at least four times and three times respectively more than the Otovent manufacturer's limit (even when looking at the ‘worst predictable balloon’ extrapolation), showing their possible effectiveness in clinical practice. Autoinflation balloons were able to be tested against spherical balloons for the first time in healthy patients allowing their effectiveness at performing a Valsalva manoeuvre and their subsequent potential use in the treatment of OME.

### Strengths and limitations

A range of party balloons were able to be tested from a variety of different manufacturers. Guidance for the use of Otovent balloons suggests that an adult inflates the balloon for the first time to show the child how to use it and to overcome the larger pressure required for the first inflation. This further supports the use of sphere party balloons as the larger pressure required to initially inflate sphere 2 (E) balloons (Table 3) could be negated by adult assistance. All balloons were tested under repeated stress that was greater than would have been experienced by normal use. Inflations for each balloon happened in quick succession compared with three inflations per day in ordinary use of Otovent balloons. This was demonstrated by a pressure increase between inflations 20 and 21 (supplementary Figure S1), as two of the three balloon types did not have the final 30 inflations carried out immediately after the first 20. This might suggest that the balloons could still achieve the required pressures for even more inflations if the intervals between inflations were longer.

The analysis considers the variability between different balloons tested, although, as the values are extrapolated, an individual balloon may burst before this point. The main limitation was the use of the nosepiece to attach all balloons to the tubing connected to the pressure monitor. If spherical party balloons in treatment were used as opposed to Otovent, then the nosepiece would not be available; this could prevent a seal being created to inflate the balloons. However, in the pilot clinical trial, participants still reported a Valsalva manoeuvre despite the absence of a nosepiece. It was not possible to blind the participants as to which balloon they were using, although this reflected how these balloons would be used in routine practice. The sample size of the pilot clinical trial was limited by the number of Otovent balloons remaining from the original data collection and, consequently, so was the power of the analysis. Therefore, it would only have been possible to detect large differences between the three groups.

The experiment did not assess the effect of different inflation speeds, or of different volumes required to attain a pressure sufficient to achieve a Valsalva manoeuvre. The equipment used inflates at a roughly constant speed, which is probably faster than that which could be achieved by a person, particularly a child.

### Comparison with existing literature

In comparison with a previous study with party balloons,^27^ the ability of specialised autoinflation balloons was compared with party balloons. Also, unlike previous experiments, the maximum pressure reached by each balloon via a pressure monitor was determined in order to assess whether a Valsalva manoeuvre could take place. Recent randomised clinical trials^28^ suggest that autoinflation is an effective way for treating OME during the watchful-waiting period and this study's results confirm the reliability of Otovent balloons to consistently reach the required pressure for a Valsalva manoeuvre.

### Implications for research and practice

The results have suggested that spherical party balloons and Otovent balloons have limited differences for patients owing to the ‘all-or-nothing’ nature of a Valsalva manoeuvre. This implies that spherical party balloons could be used instead of Otovent balloons for treatment of OME, which would provide large cost savings. Otovent balloons are sold in a pack of five and, at drug tariff prices, are £0.98 per balloon, while the spherical balloons used in this study are sold in packs of 50 and average £0.029 per balloon. The recommended treatment time for autoinflation balloons is between 1 and 3 months (during the NICE guideline recommended watchful-waiting period). Therefore, if a balloon has a maximum of 20 inflations (based on Otovent instructions) and is to be used three times per day, five balloons would be required per month of treatment. For 1 month, the cost would be £0.15 (spherical) compared with £4.90 (Otovent), while for 3 months of treatment this would be £0.45 (spherical) compared with £14.70 (Otovent). Further savings could be obtained if a larger number of inflations were used before discarding a balloon, which the data indicates may be possible.

Further experiments need to be carried out in a randomised control trial to compare the use of spherical party balloons with specialised Otovent balloons on a paediatric patient group with OME, to test compliance as well as effectiveness. Otovent balloons have been shown to have a compliance rate of 80% at 3 months.^18^ It would also be important to test whether or not a nosepiece is necessary to create the seal required to reach the necessary pressure to perform a Valsalva manoeuvre, especially in a young child under supervision. An alternative could be to use a nosepiece initially and then transition to using party balloons with or without the original nosepiece. Further research is also needed to test whether there is an infection risk of repeatedly using any balloon type beyond the 20 inflation Otovent manufacturer’s limit.
